# Single or combined ablation of peripheral serotonin and p21 limit adipose tissue expansion and metabolic alterations in early adulthood in mice fed a normocaloric diet

**DOI:** 10.1371/journal.pone.0255687

**Published:** 2021-08-11

**Authors:** Enrica Saponara, Rong Chen, Theresia Reding, Richard Zuellig, Darren C. Henstridge, Rolf Graf, Sabrina Sonda

**Affiliations:** 1 Department of Visceral and Transplantation Surgery, Swiss Hepato-Pancreato-Biliary Center, University Hospital Zurich, Zurich, Switzerland; 2 Division of Endocrinology, Diabetes & Clinical Nutrition, University Hospital Zurich, Zurich, Switzerland; 3 School of Health Sciences, College of Health and Medicine, University of Tasmania, Launceston, TAS, Australia; 4 Zurich Center for Integrative Human Physiology (ZIHP), University of Zurich, Zurich, Switzerland; Medical University of Vienna, AUSTRIA

## Abstract

Identifying the fundamental molecular factors that drive weight gain even in the absence of hypercaloric food intake, is crucial to enable development of novel treatments for the global pandemic of obesity. Here we investigated both adipose tissue-specific and systemic events that underlie the physiological weight gain occurring during early adulthood in mice fed a normocaloric diet. In addition, we used three different genetic models to identify molecular factors that promote physiological weight gain during normocaloric and hypercaloric diets. We demonstrated that normal physiological weight gain was accompanied by an increase in adipose tissue mass and the presence of cellular and metabolic signatures typically found during obesity, including adipocyte hypertrophy, macrophage recruitment into visceral fat and perturbed glucose metabolism. At the molecular level, this was associated with an increase in adipose tissue tryptophan hydroxylase 1 (Tph1) transcripts, the key enzyme responsible for the synthesis of peripheral serotonin. Genetic inactivation of Tph1 was sufficient to limit adipose tissue expansion and associated metabolic alterations. Mechanistically, we discovered that Tph1 inactivation resulted in down-regulation of cyclin-dependent kinase inhibitor p21^Waf1/Cip1^ expression. Single or double ablation of Tph1 and p21 were equally effective in preventing adipocyte expansion and systemic perturbation of glucose metabolism, upon both normocaloric and hypercaloric diets. Our results suggest that serotonin and p21 act as a central molecular determinant of weight gain and associated metabolic alterations, and highlights the potential of targeting these molecules as a pharmacologic approach to prevent the development of obesity.

## Introduction

Accumulation of excessive body fat is a growing public health problem, as the number of people overweight or obese has tripled in the last 40 years, affecting more than two in three adults. Alarmingly, the problem has reached global proportions, concerning both developed and emerging countries [1], and all age groups [2, 3] (WHO statistics https://www.who.int/en/news-room/fact-sheets/detail/obesity-and-overweight).

Obesity is associated with the development of numerous life-threatening comorbidities, including type 2 diabetes, dyslipidemia, hypertension, non-alcoholic fatty liver disease and cardiovascular diseases [4], which are responsible for 70% of deaths worldwide [5, 6]. In this context, finding effective and safe approaches to prevent and resolve obesity is of paramount importance to improve quality of life in both present and future generations.

Development of obesity is the result of a multifaceted interaction between different elements, including eating behaviour, sedentary life-styles, psychological factors, gut microbiota and genetic components (reviewed in [7]. Importantly, the mainstay anti-obesity approaches of hypocaloric diet and physical exercise have been proven insufficient to resolve the disease in the long term in the majority of individuals [8, 9]. Thus, new pharmacologic interventions are needed to complement the existing therapy and require elucidating the molecular determinants of weight gain.

The monoamine signalling molecule serotonin (5-hydroxytryptamine, 5-HT) is a potent regulator of energy balance, acting both at the level of central nervous system (CNS) and in the periphery. While the predominant effect of serotonin in the CNS is the suppression of food intake, the molecule exerts an opposing function in the periphery, negatively influencing glucose homeostasis and lipid metabolism (reviewed in [10]) and promoting the development of obesity upon hypercaloric food intake [11, 12]. This dual role of serotonin is supported by the fact that the molecule is synthesised by two different rate-limiting enzymes, physically compartmentalised in the CNS (tryptophan hydroxylase 2, Tph2) and in the periphery (tryptophan hydroxylase 1, Tph1). Important for anti-obesity interventions, genetic or pharmacologic inhibition of Tph1 or serotonin receptors limits weight gain induced by high fat diet [12–14].

While these findings reveal a prominent role of serotonin in driving weight gain induced by hypercaloric food intake, it is not known whether serotonin controls weight gain in the absence of obesogenic diets. To answer this question, we characterized the physiological weight gain that occurs in early adulthood, when normal levels of body fat percentage and average waist circumference increase in both men and women, according to normative reference data [15–17].

The objectives of this study were twofold. Firstly, to test whether this physiological weight gain is associated with metabolic changes that may predispose to the development of metabolic dysfunctions later in life. Secondly, to analyse whether serotonin and its downstream effectors are the molecular regulators of physiological weight gain, which may be targeted for preventive or therapeutic interventions to counteract obesity.

Mice in the early phase of adulthood were used to examine the regulation of body weight and metabolic parameters. This experimental model is a relevant approach as i) body weight steadily increases in mice from adolescence through middle age [18, 19]; ii) similar to what is observed in humans, increased body weight during early adulthood is matched by an increase in fat mass [19]; iii) increased body weight is observed upon feeding a normocaloric diet and consistent food intake [18].

## Materials and methods

### Reagents

Unless otherwise stated, all chemicals were purchased from Sigma-Aldrich, Buchs, Switzerland. Cell culture reagents were from Gibco-BRL, Basel, Switzerland. Reagent stock solutions were freshly diluted to the concentrations required for the individual experiment indicated in the result section.

### Animal experiments

All animal experiments were conducted in accordance with Swiss federal animal regulations and approved by the cantonal veterinary office of Zurich. All studies involving animals are reported in accordance with the ARRIVE and MINPEPA guidelines and were designed to standardize animal handling, tissue harvesting and tissue processing. Mice were kept in individually ventilated cages with environmental enrichments; floors were covered with wood-shavings. Maximum of 5 mice were kept in a cage. Cages were maintained in a semi-natural 12:12 hours light:dark cycle and animals had free access to water and standard chow diet (3436, Kliba Nafag, Kaiseraugst, Switzerland). All animal groups consisted of healthy, ≥25 g adult male mice.

Animals used in the study were adult male mice of the following strains: inbred C57BL/6 mice (Envigo (Itingen, Switzerland), mixed background *Tph1*^-/-^, p21^-/-^, *Tph1*^-/-^/*p21*^-/-^, and wild type mice generated by crossing *Tph1*^-/-^ mice (C57BL/6 background) [20] and *p21*^-/-^ mice (B6;129S2-Cdkn1a<tm1Tyj>/J; Jackson Laboratories, USA) bred in our facility.

In the high fat diet (HFD) experiments, 2 months old mice were fed ad libitum with a diet containing 60% fat by calories (E15742, ssniff; Soest, Gemany) for 4 weeks. Body weight and blood glucose level were quantified weekly after an overnight fast. No animal died before the experiments were concluded.

### Whole body composition

Whole body composition (fat and fat free mass) was measured by nuclear magnetic resonance imaging (echoMRI tissue composition analyser, EchoMedical Systems, Houston, USA) as previously described [21], at 2 and 6 months of age. Before the measurement, mice where weighed and anesthetised with isoflurane inhalation. All measurements were performed during the light phase (9am-5pm).

### Pathology data information

Experimental pathology data have been collected in accordance with the MINPEPA guidelines, to maximise the consistency and reproducibility of results. Experiments were designed to standardize animal handling, tissue harvesting and tissue processing. Animals were harvested according to a standard operation procedure where mice were anesthetized by isoflurane inhalation, received a midline laparotomy under continuing inhalation anaesthesia, followed by dissecting and snap freezing a small piece of the pancreas near the spleen for subsequent RNA extraction. Blood was sampled by exsanguination via heart puncture, and the remaining organs were harvested for histology.

### Biochemical analyses

Glucose, insulin and pyruvate tolerance tests were performed after an overnight fast (∼15 h) or morning fast (3 h) with free access to water. Mice were weighed before an intra-peritoneal injection of glucose (2 g/kg, Sigma-Aldrich), insulin (0.75 U / kg, Novo Nordisk, Denmark) or pyruvate (2 g/kg, Sigma-Aldrich), respectively. Blood samples from live animals were collected from the tail tip and blood glucose level was measured with a glucose meter (Accu-Chek, Roche, Switzerland) at regular intervals as indicated in the figure legends.

Levels of total cholesterol (free cholesterol + cholesterol esters) and triglycerides present in serum were measured using the Fuji Dri-Chem 4000i analyzer (FUJIFILM Corporation, Tokyo, Japan). Blood was collected from the harvested animals via heart puncture.

### Immunohistochemistry

Standard procedures were used to fix tissues by immersion in 4% formalin for 24 h, dehydrate them through a series of graded ethanol baths using a Leica TP1020 Histokinette (Leica Instruments GmbH, Nussloch, Germany) and subsequently embed them in paraffin. Consecutive 3 μm-thick sections were prepared for haematoxylin-eosin (H&E) staining and immunohistochemistry [22]. Primary antibodies used in this study were rat anti-F4/80 (1:400, #T-2006 BMA Biomedicals, Augst, Switzerland) followed by rabbit anti-rat Immunoglobulins/HRP (DAB) P0450 (Agilent Technologies, Basel). All staining was performed with a DAKO autostainer Link 48 (Glostrup, Denmark).

Microscopy analyses were performed on a wide-field Nikon Eclipse Ti (Amsterdam, The Netherlands). Quantification of labelled cells was performed in at least ten randomly selected high-power fields (×200) per slide using the NIS Elements BR Analysis (Nikon, Amsterdam, The Netherlands) and Cell^P (Olympus, Tokyo, Japan) analysis software. Number of positive cells was normalized on the total tissue area present in each power field. Size of adipocytes was quantified with Adiposoft software (ImageJ, University of Navarra, USA).

### Transcript analysis

Total RNA was extracted from epididymal adipose tissue, using the RNeasy Mini Kit (Qiagen, Hilden, Germany), and reverse-transcribed with qScript™ cDNA SuperMix (Quanta Biosciences). Gene expression was measured by real-time PCR on a 7500 Fast Real-Time PCR System (Applied Biosystems, Thermo Fisher Scientific) using Taqman probes (Applied Biosystems). Transcript levels were normalized using 18S RNA as a reference and expressed as ΔΔCt relative to the value of control cells or animals. Taqman probes used in this study were: p21 (Mm00432448_m1), p27 (Mm00438168_m1), p15 (Mm00483241_m1), p16 (Mm00494449_m1), p18 (Mm00483243_m1), F480 (Mm00802530_m1), TNFα (Mm00802530_m1), Mcp1 (Mm00441242_m1), Ccl5 (Mm01302427_m1) and TaqMan^TM^ ribosomal RNA control reagent (#4308329).

### Statistics

At least five animals were tested for each experimental group. The data are expressed as means ± SEM. The statistical significance of differences in the means of experimental groups was determined using an unpaired, two-tailed Student’s *t* test or one-way analysis of variance followed by Dunnett post-test (GraphPad Prism 7; GraphPad Software, Inc.). A probability value of <0.05 was considered statistically significant.

## Results

### Increased body weight during early adulthood is accompanied by hypertrophy and inflammation of visceral adipose tissue

**Fig 1:**
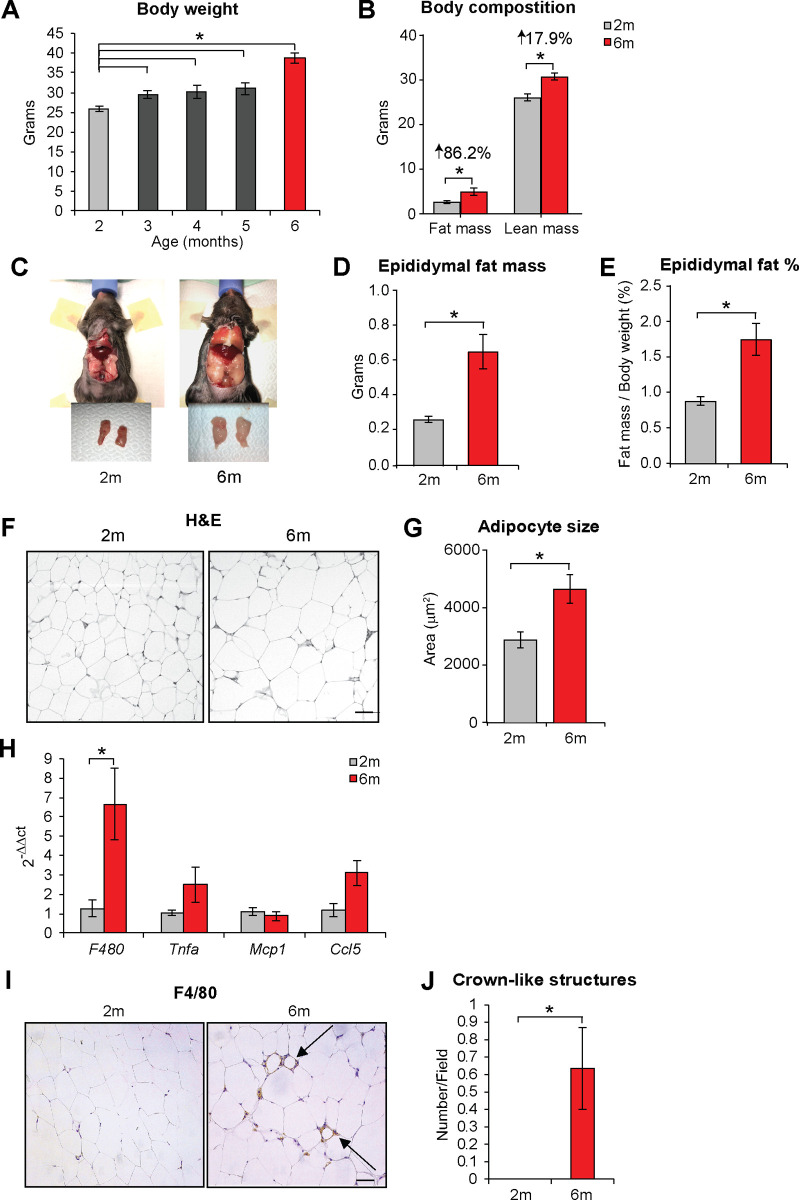
Increased body weight during early adulthood is accompanied by hypertrophy and inflammation of visceral adipose tissue. **(A)** Body weight of mice in the first 6 months of age when fed a normocaloric chow diet. **(B)** Whole body composition (fat and fat free mass) measured by nuclear magnetic resonance imaging in 2 and 6 months old mice. Numbers above the bars represent the percentage of weight increase at 6 months of age. **(C)** Representative macrograph of epididymal fat pads in 2 and 6 months old mice. **(D)** Quantification of epididymal fat mass, expressed in grams, in 2 and 6 months old mice. **(E)** Quantification of epididymal fat mass, expressed as percentage of body weight, in 2 and 6 months old mice. **(F)** Haematoxylin and Eosin (H&E) staining of epididymal fat in 2 and 6 months old mice. **(G)** Quantification of adipocyte size in epididymal fat in 2 and 6 months old mice. **(H)** qPCR of macrophage marker F480 and inflammatory mediators in epididymal fat in 2 and 6 months old mice. **(I)** Immunostaining with the macrophage marker F480 in epididymal fat in 2 and 6 months old mice. Arrows indicate crown-like structures present in the tissue. **(J)** Quantification of crown-like structures present in epididymal fat in 2 and 6 months old mice. Results are average ± SEM (n≥5), *p < 0.05.

To elucidate the changes associated with increased body weight in early adulthood, we analysed metabolic and molecular parameters in mice between 2 and 6 months of age, which corresponds to the equivalent period from adolescence to 30 years of age in humans (https://www.jax.org/news-and-insights/jax-blog/2017/november/when-are-mice-considered-old.). Body weight of male mice fed a standard normocaloric chow diet significantly increased (Fig 1A), while food intake remained comparable (S1A Fig), consistent with previous reports [18]. The increase in body weight was due to a combination of increased fat mass and lean mass, as determined by body composition analyses (Fig 1B). While lean mass contributed the highest absolute gain, fat mass had the highest relative expansion, and almost doubled at 6 months of age.

Increased body weight correlated with higher levels of cholesterol circulating in blood, while levels of triglycerides did not increase in 6 months old mice (S1B Fig). In addition, epididymal fat pads, a major reservoir of visceral adipose tissue with key metabolic roles (reviewed in [23]), significantly expanded in this time period, as seen by increased absolute (Fig 1C and 1D), and relative mass over total body weight (Fig 1E). Average size of epididymal adipocytes was also enlarged (Fig 1F and 1G), indicating that hypertrophy of adipose tissue occurs during early adulthood. Increased size and hypertrophic growth are considered a stress factor for adipocytes and a strong predictor of inflammation and macrophage infiltration during obesity [24]. Surprisingly, we found that 6 months old mice showed sign of inflammation in epididymal fat, including recruitment of macrophages (Fig 1H), crown-like structures, which represent macrophages recruited around dead adipocytes (Fig 1I and 1J), and increased trend of cytokine expression (Fig 1H).

Collectively, these data indicate that increased body weight established during early adulthood is associated with hypertrophy and inflammation of visceral adipose tissue.

### Increased body weight during early adulthood is accompanied by altered glucose metabolism

**Fig 2:**
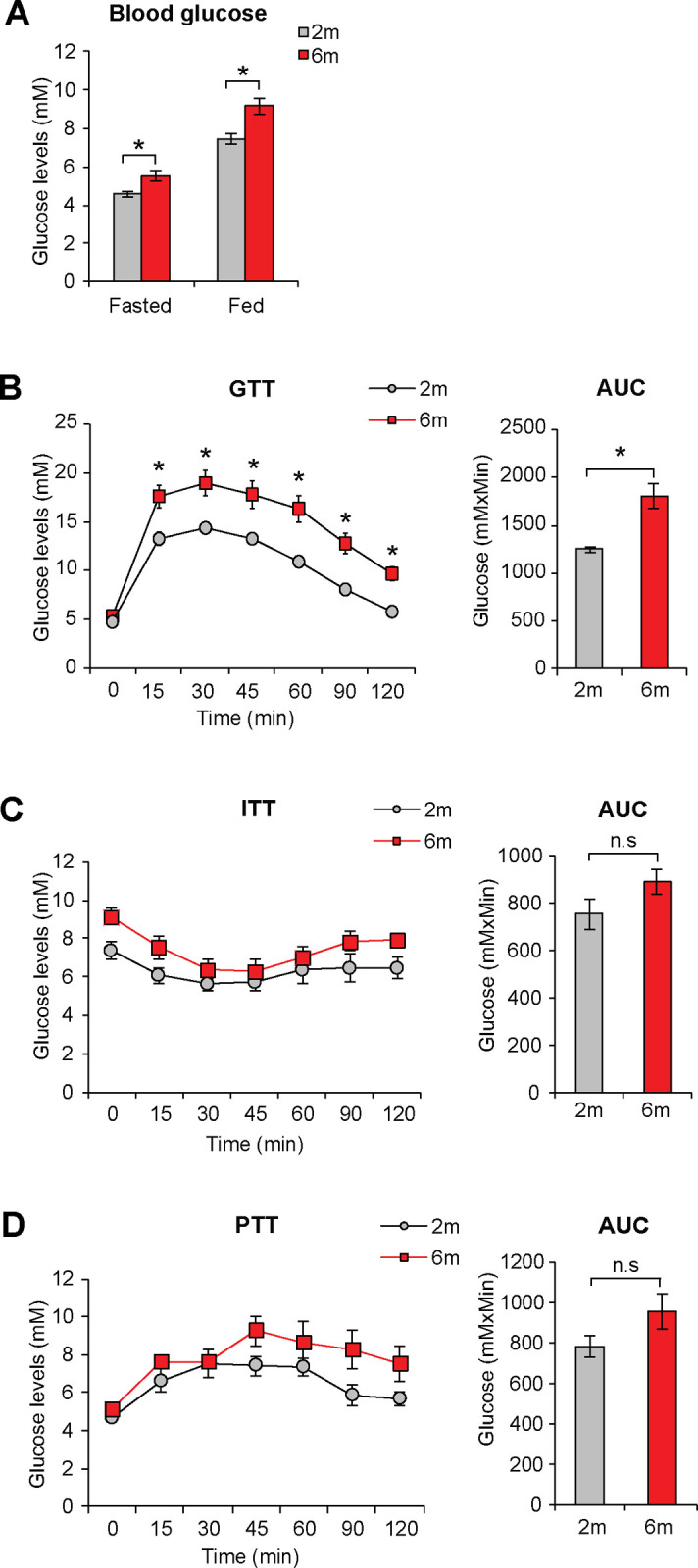
Alteration of glucose metabolism during early adulthood. **(A)** Blood glucose quantification in 2 and 6 months old mice measured in overnight fasted and fed state. **(B)** Glucose tolerance test (GTT) and area under the curve (AUC) quantification in 2 and 6 months old mice. **(C)** Insulin tolerance test (ITT) and area under the curve (AUC) quantification in 2 and 6 months old mice. **(D)** Pyruvate tolerance test (PTT) and area under the curve (AUC) quantification in 2 and 6 months old mice. Results are average ± SEM (n≥5), *p < 0.05.

During obesity, visceral adipose tissue alterations, including the development of low level inflammation, are strongly associated with deterioration of glucose homeostasis, resulting in insulin sensitivity and high glycemia [25, 26]. We found that, during physiological weight gain, epididymal fat pad alterations were also accompanied by altered glucose metabolism, characterised by higher blood levels of glucose, both in the fasted and fed state (Fig 2A) and lower glucose tolerance after glucose loading during a glucose tolerance test (GTT) (Fig 2B). Glucose levels during an insulin tolerance test (ITT) or a pyruvate tolerance test (PTT) were not significantly different between the two age groups (Fig 2C and 2D), suggesting that older mice did not develop insulin resistance or altered hepatic gluconeogenesis.

### Metabolic changes in early adulthood are reduced upon genetic deletion of *Tph1*

**Fig 3:**
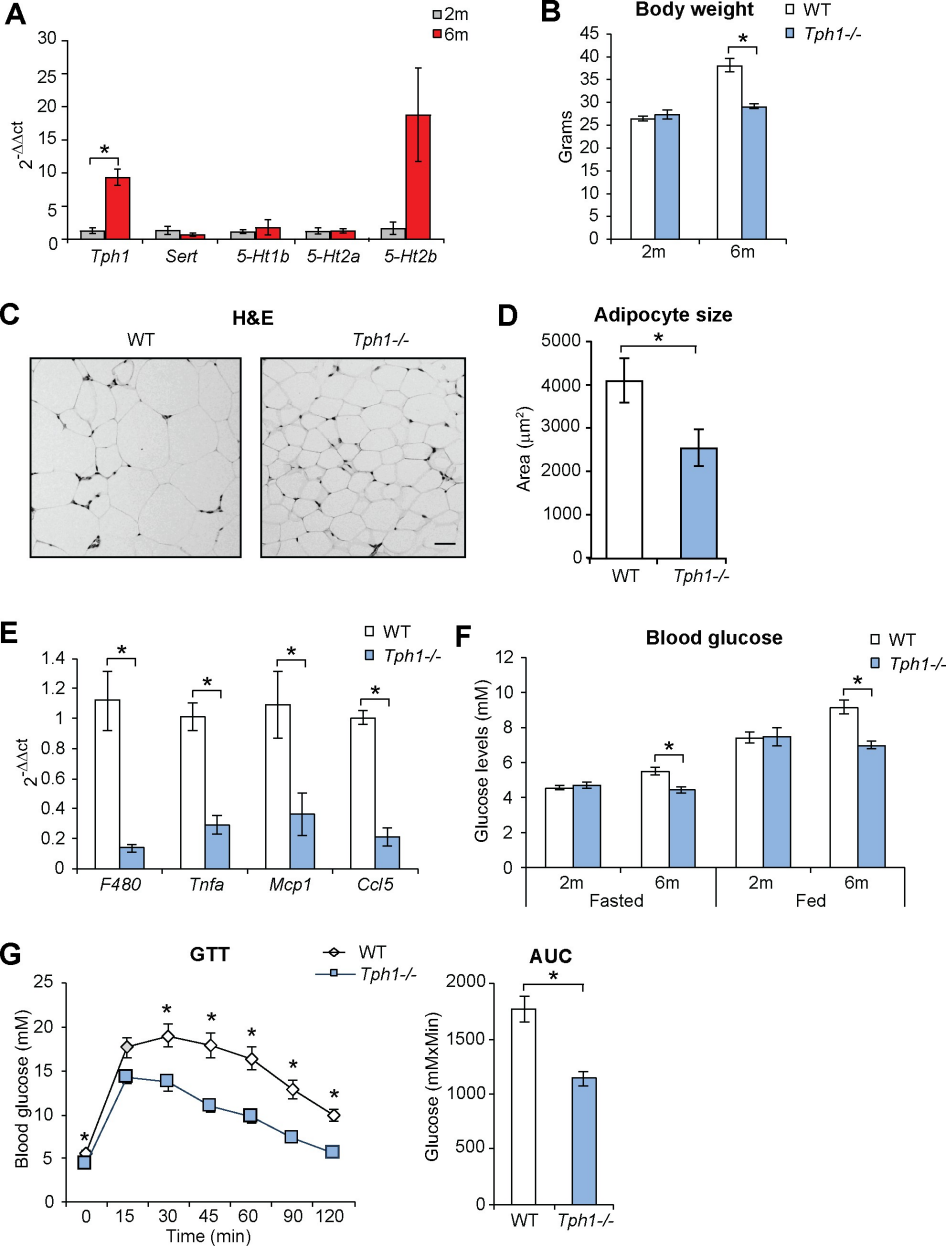
Early adulthood metabolic changes are reduced in the absence of *Tph1*. **(A)** qPCR of serotonin synthetic enzyme (*Tph1*), serotonin transporter (*Sert*), serotonin receptors (*5-Ht1b*, *5-Ht2a*, *5-Ht2b*) in epididymal fat in 2 and 6 months old mice. **(B)** Body weight of wild type (WT) and *Tph1* deficient (*Tph1*^*-/-*^) mice at 2 and 6 months of age. **(C)** Haematoxylin and Eosin (H&E) staining of epididymal fat in WT and *Tph1*^*-/-*^ mice at 6 months of age. **(D)** Quantification of adipocyte size in epididymal fat in 6 months old mice. **(E)** qPCR of macrophage marker F480 and inflammatory mediators in epididymal fat in 6 months old mice. **(F)** Blood glucose quantification in 2 and 6 months old mice measured in overnight fasted and fed state. **(G)** Glucose tolerance test (GTT) and area under the curve (AUC) quantification in 6 months old mice. Results are average ± SEM (n≥5), *p < 0.05.

Increased expression of *Tph1* in adipose tissue and consequent increased serotonin levels in the periphery promote weight gain during consumption of high fat diet [12, 13]. Thus, we tested whether increased *Tph1* levels are also observed upon physiologic weight gain during early adulthood and normocaloric diet. Expression of *Tph1* transcripts increased in epididymal fat pads of 6 months old mice, while levels of serotonin transporter (*Sert*) and serotonin receptor isoforms *5-Ht1b*, *5-Ht2a* and *5-Ht2b* did not significantly change with time (Fig 3A).

To test whether the time-dependent increase in *Tph1* expression is responsible for the metabolic changes observed during early adulthood, we compared lipid and glucose parameters at 2 and 6 months of age in wild type and *Tph1* knocked out mice (*Tph1*^*-/-*^), which are characterised by reduced levels of serotonin in the periphery [20] and which we previously used to demonstrate the role of serotonin in pancreatic pathophysiology [27–29].

Body weight was comparable in the two strains at 2 months of age, as previously shown [13], however, it was lower in *Tph1*^*-/-*^ mice at 6 months of age (Fig 3B). Blood levels of cholesterol and triglycerides were lower in *Tph1*^*-/-*^ mice at all time points analysed (S2A Fig). Adipocytes were smaller in epididymal fat pads of 6 months old Tph1^-/-^ mice (Fig 3C and 3D), together with reduced expression of macrophage markers and inflammatory factors (Fig 3E).

Blood levels of glucose were also lower in older *Tph1*^*-/-*^ mice, both in the fasted and fed state (Fig 3F). Similarly, glucose tolerance was better in older *Tph1*^*-/-*^ mice compared to wild type animals (Fig 3G), while insulin tolerance and pyruvate tolerance did not change in the two strains (S2B and S2C Fig).

Collectively, our results show that ablation of *Tph1*^*-/-*^ limits the metabolic changes observed during early adulthood.

### Metabolic changes in early adulthood are reduced upon single or combined genetic deletion of *Tph1* and *p21*

**Fig 4:**
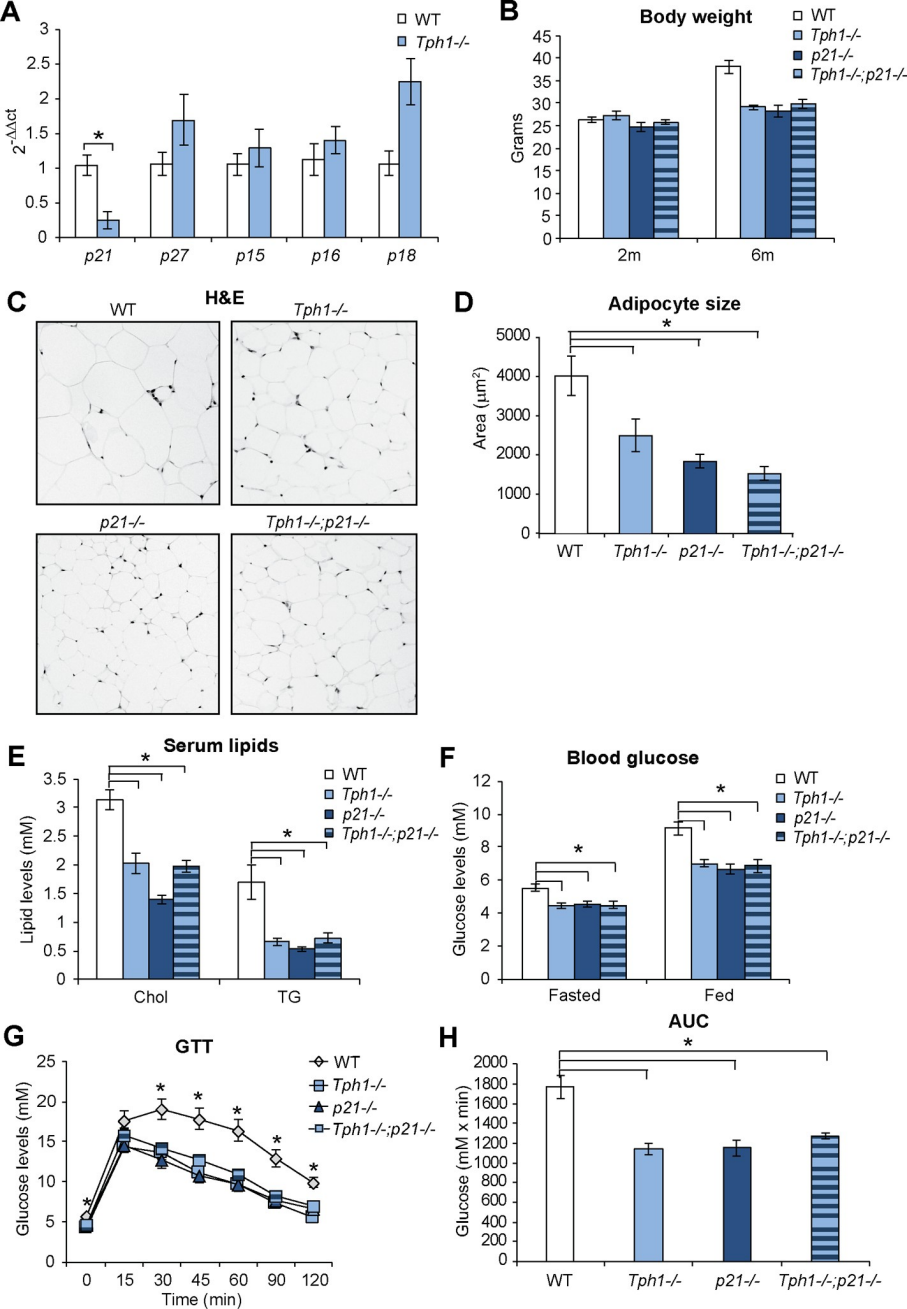
Early adulthood metabolic changes are reduced in the absence of *p21*. **(A)** qPCR of cell cycle inhibitors in epididymal fat in 6 months old wild type (WT) and *Tph1* deficient (*Tph1*^*-/-*^) mice. **(B)** Quantification of body weight of WT, *Tph1*^*-/-*^, *p21* deficient (*p21*^*-/-*^) and *Tph1*, *p21* double deficient (*Tph1*^*-/-*^;*p21*^*-/-*^) mice at 2 and 6 months of age. **(C)** Haematoxylin and Eosin (H&E) staining of epididymal fat in WT, *Tph1*^*-/-*^, *p21*^*-/-*^ and *Tph1*^*-/-*^;*p21*^*-/-*^ mice at 6 months of age. **(D)** Quantification of adipocyte size in epididymal fat in 6 months old mice. **(E)** Quantification of blood cholesterol (Chol) and triglyceride (TG) levels in WT, *Tph1*^*-/-*^, *p21*^*-/-*^ and *Tph1*^*-/-*^;*p21*^*-/-*^ mice at 6 months of age. **(F)** Blood glucose levels in WT, *Tph1*^*-/-*^, *p21*^*-/-*^ and *Tph1*^*-/-*^;*p21*^*-/-*^ mice at 6 months of age in fasted and fed state. **(G)** Glucose tolerance test (GTT) in WT, *Tph1*^*-/-*^, *p21*^*-/-*^ and *Tph1*^*-/-*^;*p21*^*-/-*^ mice at 6 months of age. **(H)** Area under the curve (AUC) of GTT in WT, *Tph1*^*-/-*^, *p21*^*-/-*^ and *Tph1*^*-/-*^;*p21*^*-/-*^ mice at 6 months of age.Results are average ± SEM (n≥5), *p < 0.05.

We then investigated whether the smaller epididymal adipocytes in *Tph1*^*-/-*^ mice were accompanied by altered expression of factors that regulate adipocyte size. Increased adipocyte size is promoted by up-regulation of the cyclin-dependent kinase inhibitor p21^Waf1/Cip1^ during the process of adipocyte formation, [30]. We found that expression of *p21* transcripts was reduced in epididymal fat pads of *Tph1*^*-/-*^ mice, while expression of other cyclin-dependent kinase inhibitors did not decrease (Fig 4A).

To further investigate whether activation of Tph1 and p21 signalling mediates the metabolic changes observed during early adulthood, we tested whether single ablation of *p21*, or combined with *Tph1* ablation, limited weight gain and glucose alterations. Body weight was comparable in the four strains at 2 months of age, whereas it was lower in all knocked out mice at 6 months of age (Fig 4B). Daily food intake showed a reduced trend in 6 months old KO animals, however the reduction did not reach statistical significance (S3A Fig). In addition, daily food intake normalised on body weight was comparable in the 4 strains analysed (S3B Fig). Adipocyte size of epididymal fat pads was smaller upon single or combined deletion of *p21* and *Tph1*, as shown by histological examination (Fig 4C) and cell area quantification (Fig 4D). Moreover, levels of circulating cholesterol and triglycerides were lower in knocked out mice (Fig 4E). This was paralleled by better glucose homeostasis, with lower levels of circulating glucose, in both the fasted and fed states (Fig 4F), and better glucose tolerance (Fig 4G and 4H). Collectively, our results show that ablation of either *Tph1* or *p21* limits the metabolic changes observed upon weight gain during early adulthood. In addition, combined ablation of the two genes did not provide a cumulative change in metabolic parameters, suggesting that disruption of either factor (*Tph1* or *p21*) is sufficient for the maximal effect.

### Ablation of *Tph1* or *p21* limits early adulthood-associated fat mass accumulation and prevents high fat diet-induced weight gain

**Fig 5:**
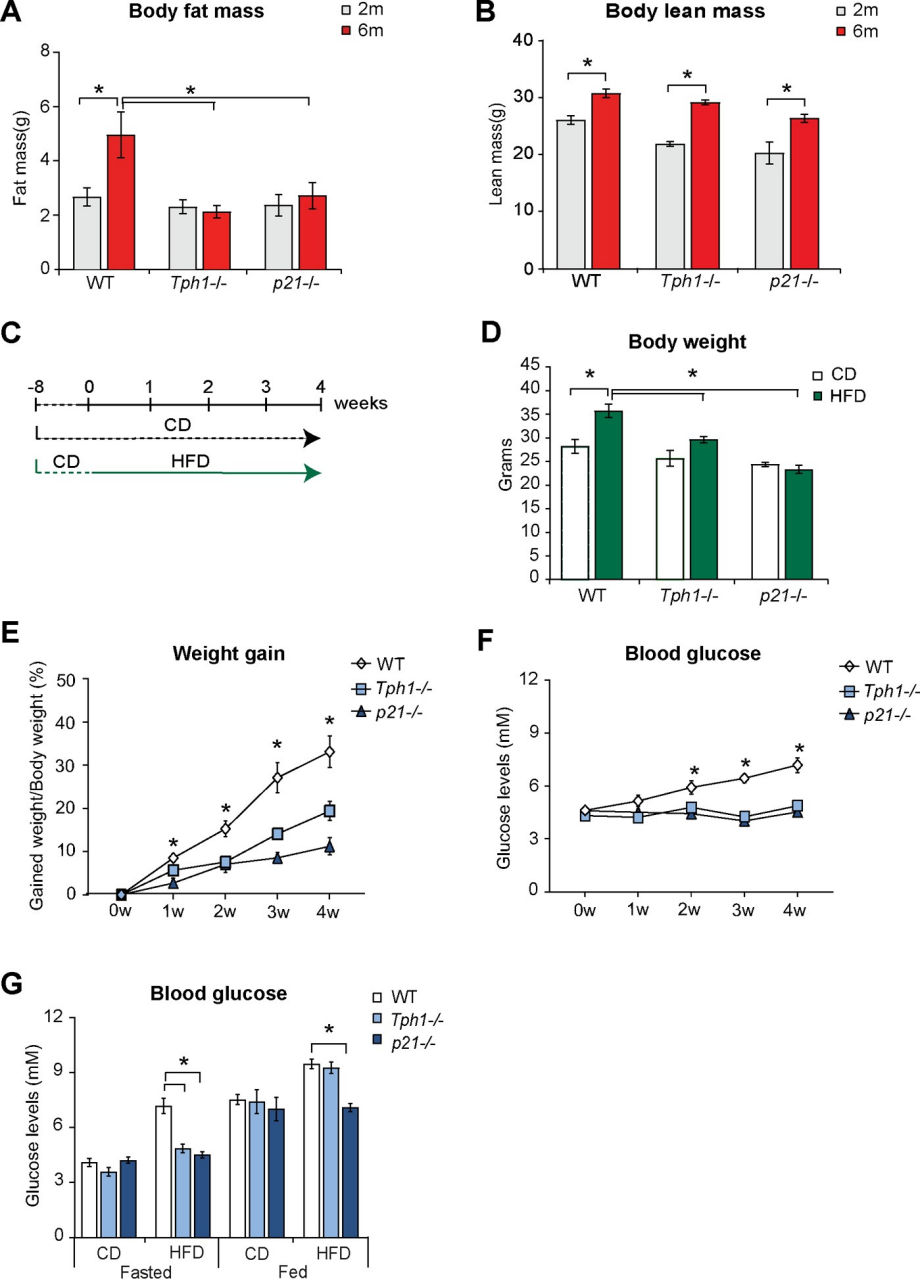
Ablation of *Tph1* or *p21* limits early adulthood-associated fat mass accumulation and prevents high fat diet-induced weight gain. **(A)** EchoMRI quantification of whole body fat mass in wild type (WT), *Tph1* deficient (*Tph1*^*-/-*^) and *p21* deficient (*p21*^*-/-*^) mice at 2 and 6 months of age. **(B)** EchoMRI quantification of whole body lean mass in WT, *Tph1*^*-/-*^ and *p21*^*-/-*^ mice at 2 and 6 months of age. **(C)** Schematic representation of diet regimens using normocaloric standard chow diet (CD) and hypercaloric high fat diet (HFD). **(D)** Body weight of WT, *Tph1*^*-/-*^ and *p21*^*-/-*^ mice fed CD or HFD for 4 weeks. **(E)** Body weight gain, expressed as percentage of initial body weight, of WT, *Tph1*^*-/-*^ and *p21*^*-/-*^ mice during 4 weeks of HFD. **(F)** Blood glucose levels of WT, *Tph1*^*-/-*^ and *p21*^*-/-*^ mice during 4 weeks of HFD. **(G)** Comparison of blood glucose levels in fasted or fed state of WT, *Tph1*^*-/-*^ and *p21*^*-/-*^ mice after 4 weeks of CD or HFD. Results are average ± SEM (n≥5), *p < 0.05.

We further investigated whether ablation of *Tph1* or *p21* differently impacts on the mass of body components. Whole-body composition analysis performed by magnetic resonance imaging revealed that total fat mass increased in wild type but not in *Tph1*^*-/-*^ and *p21*^*-/-*^ animals from 2 to 6 months of age (Fig 5A). In contrast, lean mass increased in all strains over time (Fig 5B), suggesting that Tph1 and p21 mainly regulate the adipose tissue.

Finally, we tested whether the protective effect observed upon deletion of *Tph1* and *p21* during physiological weight gain was also evident during high fat diet (HFD)-induced weight gain. To this aim, we fed 2 months old mice with HFD for 4 weeks (Fig 5C). We chose this short-term exposure to HFD to capture the metabolic changes induced by high caloric food consumption and limit the confounding influence of age-induced changes. HFD increased the body weight of wild type mice, compared to a standard normocaloric diet (Fig 5D). However, increase of body weight was limited in *Tph1*^*-/-*^ or *p21*^*-/-*^ mice, evident by comparing the weight of mice fed a normocaloric or HFD (Fig 5D) and by quantifying the percentage of weekly weight gain in during HFD (Fig 5E).

As previously observed during spontaneous weight gain, HFD-induced weight gain correlated with a progressive increase in the levels of circulating glucose, which was limited in the absence of *Tph1* or *p21* (Fig 5F), especially evident when animals where in a fasted state (Fig 5G).

Collectively, these results indicate that *Tph1* and *p21* regulate metabolic parameters during physiological and diet-induced weight gain.

## Discussion

The most effective approach to develop prevention- and intervention-based strategies to counteract the obesity pandemic requires a combination of life style changes together with pharmacological targeting of key molecular factors that promote the accumulation of fat mass. To identify these factors, we investigated the determinants of weight gain that occurs spontaneously during the early phases of adulthood. The analysis of this physiological process allowed us to identify the metabolic and molecular signatures intrinsically associated with increased fat mass that are independent from obesogenic life-style choices, including hypercaloric food intake. In addition, these signatures are also largely independent from changes in physical activity, as this parameter remains constant in mice between the age groups utilised in this study [31].

We found that adult mice had a preferential relative expansion of fat mass between the age of 2 and 6 months, which corresponds to up to 30 years of age in humans. Notably, visceral fat depots significantly increased in this time frame, bearing important implications for potential health-related problems, as increased visceral fat is responsible for the metabolic consequences of obesity [32, 33]. Surprisingly, expansion of visceral fat mass was associated with enlargement of adipose cells and recruitment of macrophages. These alterations are considered diagnostic features of adipose tissue dysfunction and are thought to be causally linked, with enlargement of adipocytes leading to cellular stress and low-grade, chronic inflammation (reviewed in [34]). An additional key sign of adipocyte dysfunction is the appearance of crown-like structures, constituted by macrophages surrounding dead adipocytes [35]. Importantly, crown-like structures are highly prevalent in visceral fat depots during obesity, both in mice and humans. They are also associated with altered glucose metabolism and the development of insulin resistance [36, 37]. Thus, the triad of increased adipocyte size, crown-like structures and development of insulin resistance represent a central element in the progression of metabolic dysfunctions associated with obesity. Our data showed that 6 month old mice not only presented crown-like structures in visceral fat depots but also had higher levels of blood glucose and lower glucose tolerance. However, blood glucose levels were below the cut-off values that are used to characterize diabetes in mice (glucose levels ≥11mM and ≥16mM in the fasted and fed state, respectively) and overt insulin resistance was not detected. This suggests that changes in visceral adipose tissue and glucose metabolism already occur during spontaneous weight gain in early adulthood without the external trigger of hypercaloric diet.

Mechanistically, we found that the serotonin signalling axis, supported by upregulation of the serotonin-synthetic enzyme Tph1, is upregulated in visceral adipose tissue and promotes weight gain and associated metabolic changes during a normocaloric diet. This discovery expands and complements previous findings demonstrating that serotonin is a key regulator of weight gain triggered by hypercaloric diet [12, 13]. These previous studies also showed that targeting peripheral serotonin synthesis is an effective approach to limit weight gain induced by high fat diet, as genetic deletion, like in our study, or pharmacologic inhibition of Tph1 protected mice from adipocyte hypertrophy and metabolic alterations. Collectively, our and previous studies identify a conserved role of serotonin in driving the expansion of adipose tissue upon normocaloric and hypercaloric diet and support the broader concept that peripheral serotonin acts as an endocrine agent stimulating energy storage. Previous studies using mice exposed to hypercaloric diet revealed that serotonin exerts a concerted effect on both white and brown adipose tissue, by stimulating lipogenesis in the former, and inhibiting thermogenesis in the latter [12, 13]. In our study using mice exposed to a normocaloric diet, we discovered that the cyclin-dependent kinase inhibitor p21 is downregulated in *Tph1* knock-out mice, and that its ablation is sufficient to limit weight gain, adipose tissue expansion and alteration of metabolic parameters during early adulthood, thus recapitulating the effects observed in the absence of peripheral serotonin. p21 is a potent inhibitor of cell cycle and it is involved in the process of adipogenesis, where adipocyte precursors undergo mitotic arrest before terminal differentiation into adipocytes [30, 38]. Thus, it is tempting to envisage a dual role of serotonin in promoting both adipocyte differentiation, by inducing p21 (our study), and adipocyte enlargement, by stimulating lipogenesis [12]. While further studies are required to verify this hypothesis, it is important to mention that the effect of p21 in regulating adipocyte expansion was also observed in mice fed a hypercaloric diet (our study and [30]), once again suggesting that serotonin and p21 are components of a conserved mechanism activated during any type of weight gain. Contrary to these finding, Khanna reported increased weight and susceptibility to high fat-induced atherosclerosis in p21 deficient mice [39]. Further studies are required to understand whether the different phenotypes observed depend on different parameters reported in the studies, including genetic background of the animals, length of diet administration, and diet composition.

Future studies are also required to quantify the level of spontaneous physical activity in our genetic models, as a change in behaviour may impact on the observed phenotype. However, pharmacologic inhibition or genetic deletion of Tph did not change the physical activity of mice [12, 13].

## Conclusions

Our data demonstrate that activation of the serotonin signalling axis in the periphery constitutes a necessary underlying mechanism of weight gain and adipose tissue expansion that occurs in early adulthood. Importantly, this physiological weight gain presents metabolic and cellular signatures similar to the ones associated with overweight and obesity. This suggests that obesogenic life-styles, including excessive consumption of hypercaloric diet and sedentary behaviour, amplify existing physiological processes rather than trigger new events that lead to cellular dysfunction and systemic alteration. Thus, targeting serotonin signalling may constitute an effective strategy to control the expansion of adipose tissue and prevent the development of associated metabolic diseases. In this context, future studies are warranted to test whether sex influences the way serotonin regulates metabolism, as fluctuating oestrogen levels can influence serotonin activity [40] and serotonin availability modulates the level of circulating oestrogen [41]. Additional investigations to elucidate the components of the serotonin signalling that regulate its effects on metabolism are key to fully elucidate the mechanisms of serotonin action and to discover novel therapeutic targets. While different serotonin receptors have been identified as critical mediators of metabolic changes in several organs (reviewed in [42]), the serotonin transporter SERT is also emerging as a pivotal player in the regulation of metabolism mediated by serotonin signalling, and SERT reduction or absence associates with metabolic syndrome and obesity [43–46]. Further studies using tissue-specific SERT KO models will reveal whether SERT effect is mainly due to a regulation of circulating serotonin levels or modulation of intracellular serotonin levels and protein serotonylation [47, 48].

## Supporting information

S1 Fig (A) Daily amount of food consumed per mouse at 2 and 6 months of age. (B) Quantification of blood cholesterol (Chol) and triglyceride (TG) levels at 2 and 6 months of age. Results are average ± SEM (n≥5), *p < 0.05.
(DOCX)

S2 Fig (A) Quantification of blood cholesterol (Chol) and triglyceride (TG) levels in wild type (WT) and Tph1 deficient (Tph1-/-) mice at 2 and 6 months of age. (B) Insulin tolerance test (ITT) in WT and Tph1-/- mice at 6 months of age. (C) Pyruvate tolerance test (PTT) in WT and Tph1-/- mice at 6 months of age. Results are average ± SEM (n≥5), *p < 0.05.
(DOCX)

S3 Fig (A) Daily amount of food consumed per mouse in wild type (WT), Tph1 deficient (Tph1-/-), p21 deficient (p21-/-), and combined Tph1 and p21 deficient (Tph1-/-;p21-/-) mice at 6 months of age. (B) Daily amount of food consumed per mouse normalised on body weight in WT, Tph1-/-, p21-/-, and Tph1-/-;p21-/-) mice at 6 months of age. Results are average ± SEM (n≥5).
(DOCX)

## Abbreviations

Tph1: tryptophan hydroxylase 1
H&E: haematoxylin-eosin
HFD: high fat diet
GTT: glucose tolerance test
ITT: insulin tolerance test
PTT: pyruvate tolerance test
Sert: serotonin transporter

## References

[1] Collaboration NCDRF. Worldwide trends in body-mass index, underweight, overweight, and obesity from 1975 to 2016: a pooled analysis of 2416 population-based measurement studies in 128.9 million children, adolescents, and adults. Lancet. 2017;390(10113):2627–42. doi: 10.1016/S0140-6736(17)32129-3 29029897 PMC5735219

[2] Ogden CL, Yanovski SZ, Carroll MD, Flegal KM. The epidemiology of obesity. Gastroenterology. 2007;132(6):2087–102. doi: 10.1053/j.gastro.2007.03.052 17498505

[3] Kumar S, Kelly AS. Review of Childhood Obesity: From Epidemiology, Etiology, and Comorbidities to Clinical Assessment and Treatment. Mayo Clin Proc. 2017;92(2):251–65. doi: 10.1016/j.mayocp.2016.09.017 28065514

[4] Ezzati M, Riboli E. Behavioral and dietary risk factors for noncommunicable diseases. N Engl J Med. 2013;369(10):954–64. doi: 10.1056/NEJMra1203528 24004122

[5] Collaborators GBDCoD. Global, regional, and national age-sex specific mortality for 264 causes of death, 1980–2016: a systematic analysis for the Global Burden of Disease Study 2016. Lancet. 2017;390(10100):1151–210. doi: 10.1016/S0140-6736(17)32152-9 28919116 PMC5605883

[6] Flegal KM, Kit BK, Orpana H, Graubard BI. Association of all-cause mortality with overweight and obesity using standard body mass index categories: a systematic review and meta-analysis. JAMA. 2013;309(1):71–82. doi: 10.1001/jama.2012.113905 23280227 PMC4855514

[7] McAllister EJ, Dhurandhar NV, Keith SW, Aronne LJ, Barger J, Baskin M, et al. Ten putative contributors to the obesity epidemic. Crit Rev Food Sci Nutr. 2009;49(10):868–913. doi: 10.1080/10408390903372599 19960394 PMC2932668

[8] Wing RR, Phelan S. Long-term weight loss maintenance. Am J Clin Nutr. 2005;82(1 Suppl):222S–5S. 16002825 10.1093/ajcn/82.1.222S

[9] Jeffery RW, Drewnowski A, Epstein LH, Stunkard AJ, Wilson GT, Wing RR, et al. Long-term maintenance of weight loss: current status. Health Psychol. 2000;19(1S):5–16. doi: 10.1037/0278-6133.19.suppl1.5 10709944

[10] Donovan MH, Tecott LH. Serotonin and the regulation of mammalian energy balance. Front Neurosci. 2013;7:36. doi: 10.3389/fnins.2013.00036 23543912 PMC3608917

[11] Kim HJ, Kim JH, Noh S, Hur HJ, Sung MJ, Hwang JT, et al. Metabolomic analysis of livers and serum from high-fat diet induced obese mice. J Proteome Res. 2011;10(2):722–31. doi: 10.1021/pr100892r 21047143

[12] Oh CM, Namkung J, Go Y, Shong KE, Kim K, Kim H, et al. Regulation of systemic energy homeostasis by serotonin in adipose tissues. Nat Commun. 2015;6:6794. doi: 10.1038/ncomms7794 25864946 PMC4403443

[13] Crane JD, Palanivel R, Mottillo EP, Bujak AL, Wang H, Ford RJ, et al. Inhibiting peripheral serotonin synthesis reduces obesity and metabolic dysfunction by promoting brown adipose tissue thermogenesis. Nat Med. 2015;21(2):166–72. doi: 10.1038/nm.3766 25485911 PMC5647161

[14] Shong KE, Oh CM, Namkung J, Park S, Kim H. Serotonin Regulates De Novo Lipogenesis in Adipose Tissues through Serotonin Receptor 2A. Endocrinol Metab (Seoul). 2020. 10.3803/EnM.2020.35.2.470 PMC7386107 32615731

[15] Imboden MT, Welch WA, Swartz AM, Montoye AH, Finch HW, Harber MP, et al. Reference standards for body fat measures using GE dual energy x-ray absorptiometry in Caucasian adults. PLoS One. 2017;12(4):e0175110. doi: 10.1371/journal.pone.0175110 28388669 PMC5384668

[16] He X, Li Z, Tang X, Zhang L, Wang L, He Y, et al. Age- and sex-related differences in body composition in healthy subjects aged 18 to 82 years. Medicine (Baltimore). 2018;97(25):e11152. doi: 10.1097/MD.0000000000011152 29924020 PMC6023800

[17] Ladabaum U, Mannalithara A, Myer PA, Singh G. Obesity, abdominal obesity, physical activity, and caloric intake in US adults: 1988 to 2010. Am J Med. 2014;127(8):717–27 e12. 10.1016/j.amjmed.2014.02.026 PMC4524881 24631411

[18] Starr ME, Saito H. Age-related increase in food spilling by laboratory mice may lead to significant overestimation of actual food consumption: implications for studies on dietary restriction, metabolism, and dose calculations. J Gerontol A Biol Sci Med Sci. 2012;67(10):1043–8. doi: 10.1093/gerona/gls009 22451471 PMC3437968

[19] Findeisen HM, Pearson KJ, Gizard F, Zhao Y, Qing H, Jones KL, et al. Oxidative stress accumulates in adipose tissue during aging and inhibits adipogenesis. PLoS One. 2011;6(4):e18532. doi: 10.1371/journal.pone.0018532 21533223 PMC3077372

[20] Walther DJ, Peter JU, Bashammakh S, Hortnagl H, Voits M, Fink H, et al. Synthesis of serotonin by a second tryptophan hydroxylase isoform. Science. 2003;299(5603):76. doi: 10.1126/science.1078197 12511643

[21] Lancaster GI, Henstridge DC. Body Composition and Metabolic Caging Analysis in High Fat Fed Mice. J Vis Exp. 2018(135). doi: 10.3791/57280 29889190 PMC6101377

[22] Silva A, Weber A, Bain M, Reding T, Heikenwalder M, Sonda S, et al. COX-2 is not required for the development of murine chronic pancreatitis. Am J Physiol Gastrointest Liver Physiol. 2011;300(6):G968–75. doi: 10.1152/ajpgi.00497.2010 21372163

[23] Chusyd DE, Wang D, Huffman DM, Nagy TR. Relationships between Rodent White Adipose Fat Pads and Human White Adipose Fat Depots. Front Nutr. 2016;3:10. doi: 10.3389/fnut.2016.00010 27148535 PMC4835715

[24] Weisberg SP, McCann D, Desai M, Rosenbaum M, Leibel RL, Ferrante AW Jr., Obesity is associated with macrophage accumulation in adipose tissue. J Clin Invest. 2003;112(12):1796–808. doi: 10.1172/JCI19246 14679176 PMC296995

[25] Xu H, Barnes GT, Yang Q, Tan G, Yang D, Chou CJ, et al. Chronic inflammation in fat plays a crucial role in the development of obesity-related insulin resistance. J Clin Invest. 2003;112(12):1821–30. doi: 10.1172/JCI19451 14679177 PMC296998

[26] Goossens GH. The role of adipose tissue dysfunction in the pathogenesis of obesity-related insulin resistance. Physiol Behav. 2008;94(2):206–18. doi: 10.1016/j.physbeh.2007.10.010 18037457

[27] Saponara E, Visentin M, Baschieri F, Seleznik G, Martinelli P, Esposito I, et al. Serotonin uptake is required for Rac1 activation in Kras-induced acinar-to-ductal metaplasia in the pancreas. J Pathol. 2018;246(3):352–65. doi: 10.1002/path.5147 30058725

[28] Saponara E, Grabliauskaite K, Bombardo M, Buzzi R, Silva AB, Malagola E, et al. Serotonin promotes acinar dedifferentiation following pancreatitis-induced regeneration in the adult pancreas. J Pathol. 2015;237(4):495–507. doi: 10.1002/path.4595 26235267

[29] Sonda S, Silva AB, Grabliauskaite K, Saponara E, Weber A, Jang JH, et al. Serotonin regulates amylase secretion and acinar cell damage during murine pancreatitis. Gut. 2013;62(6):890–8. doi: 10.1136/gutjnl-2011-301724 22591619

[30] Inoue N, Yahagi N, Yamamoto T, Ishikawa M, Watanabe K, Matsuzaka T, et al. Cyclin-dependent kinase inhibitor, p21WAF1/CIP1, is involved in adipocyte differentiation and hypertrophy, linking to obesity, and insulin resistance. J Biol Chem. 2008;283(30):21220–9. doi: 10.1074/jbc.M801824200 18445590 PMC3258954

[31] Hamrick MW, Ding KH, Pennington C, Chao YJ, Wu YD, Howard B, et al. Age-related loss of muscle mass and bone strength in mice is associated with a decline in physical activity and serum leptin. Bone. 2006;39(4):845–53. doi: 10.1016/j.bone.2006.04.011 16750436

[32] Wajchenberg BL. Subcutaneous and visceral adipose tissue: their relation to the metabolic syndrome. Endocr Rev. 2000;21(6):697–738. doi: 10.1210/edrv.21.6.0415 11133069

[33] Frayn KN. Visceral fat and insulin resistance—causative or correlative? Br J Nutr. 2000;83 Suppl 1:S71–7. doi: 10.1017/s0007114500000982 10889795

[34] Haczeyni F, Bell-Anderson KS, Farrell GC. Causes and mechanisms of adipocyte enlargement and adipose expansion. Obes Rev. 2018;19(3):406–20. doi: 10.1111/obr.12646 29243339

[35] Cinti S, Mitchell G, Barbatelli G, Murano I, Ceresi E, Faloia E, et al. Adipocyte death defines macrophage localization and function in adipose tissue of obese mice and humans. J Lipid Res. 2005;46(11):2347–55. doi: 10.1194/jlr.M500294-JLR200 16150820

[36] Murano I, Barbatelli G, Parisani V, Latini C, Muzzonigro G, Castellucci M, et al. Dead adipocytes, detected as crown-like structures, are prevalent in visceral fat depots of genetically obese mice. J Lipid Res. 2008;49(7):1562–8. doi: 10.1194/jlr.M800019-JLR200 18390487

[37] Boutens L, Stienstra R. Adipose tissue macrophages: going off track during obesity. Diabetologia. 2016;59(5):879–94. doi: 10.1007/s00125-016-3904-9 26940592 PMC4826424

[38] Chang E, Kim CY. Natural Products and Obesity: A Focus on the Regulation of Mitotic Clonal Expansion during Adipogenesis. Molecules. 2019;24(6). doi: 10.3390/molecules24061157 30909556 PMC6471203

[39] Khanna AK. Enhanced susceptibility of cyclin kinase inhibitor p21 knockout mice to high fat diet induced atherosclerosis. J Biomed Sci. 2009;16:66. doi: 10.1186/1423-0127-16-66 19604372 PMC2720941

[40] O’Keane V, O’Hanlon M, Webb M, Dinan T. d-fenfluramine/prolactin response throughout the menstrual cycle: evidence for an oestrogen-induced alteration. Clin Endocrinol (Oxf). 1991;34(4):289–92. doi: 10.1111/j.1365-2265.1991.tb03768.x 1879060

[41] Zha W, Ho HTB, Hu T, Hebert MF, Wang J. Serotonin transporter deficiency drives estrogen-dependent obesity and glucose intolerance. Sci Rep. 2017;7(1):1137. doi: 10.1038/s41598-017-01291-5 28442777 PMC5430688

[42] Yabut JM, Crane JD, Green AE, Keating DJ, Khan WI, Steinberg GR. Emerging Roles for Serotonin in Regulating Metabolism: New Implications for an Ancient Molecule. Endocr Rev. 2019;40(4):1092–107. doi: 10.1210/er.2018-00283 30901029 PMC6624793

[43] Chen X, Margolis KJ, Gershon MD, Schwartz GJ, Sze JY. Reduced serotonin reuptake transporter (SERT) function causes insulin resistance and hepatic steatosis independent of food intake. PLoS One. 2012;7(3):e32511. doi: 10.1371/journal.pone.0032511 22412882 PMC3297606

[44] Giannaccini G, Betti L, Palego L, Pirone A, Schmid L, Lanza M, et al. Serotonin transporter (SERT) and translocator protein (TSPO) expression in the obese ob/ob mouse. BMC Neurosci. 2011;12:18. doi: 10.1186/1471-2202-12-18 21299850 PMC3044656

[45] Veniaminova E, Cespuglio R, Chernukha I, Schmitt-Boehrer AG, Morozov S, Kalueff AV, et al. Metabolic, Molecular, and Behavioral Effects of Western Diet in Serotonin Transporter-Deficient Mice: Rescue by Heterozygosity? Front Neurosci. 2020;14:24. doi: 10.3389/fnins.2020.00024 32132889 PMC7041415

[46] Nam SB, Kim K, Kim BS, Im HJ, Lee SH, Kim SJ, et al. The Effect of Obesity on the Availabilities of Dopamine and Serotonin Transporters. Sci Rep. 2018;8(1):4924. doi: 10.1038/s41598-018-22814-8 29563547 PMC5862836

[47] Al-Zoairy R, Pedrini MT, Khan MI, Engl J, Tschoner A, Ebenbichler C, et al. Serotonin improves glucose metabolism by Serotonylation of the small GTPase Rab4 in L6 skeletal muscle cells. Diabetol Metab Syndr. 2017;9:1. doi: 10.1186/s13098-016-0201-1 28053672 PMC5209910

[48] Paulmann N, Grohmann M, Voigt JP, Bert B, Vowinckel J, Bader M, et al. Intracellular serotonin modulates insulin secretion from pancreatic beta-cells by protein serotonylation. PLoS Biol. 2009;7(10):e1000229. doi: 10.1371/journal.pbio.1000229 19859528 PMC2760755

