# Supplementary figures and images for: Single or combined ablation of peripheral serotonin and p21 limit adipose tissue expansion and metabolic alterations in early adulthood in mice fed a normocaloric diet
Source: PLoS One. 2021 Aug 11;16(8):e0255687. doi: 10.1371/journal.pone.0255687 (PMC8357085; doi:10.1371/journal.pone.0255687)

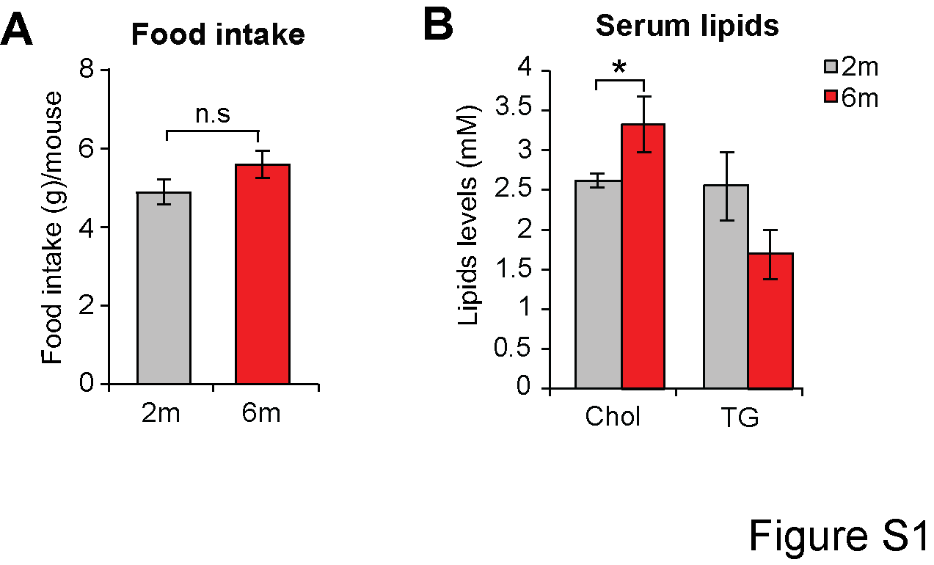

Supplement: S1 Fig — (A) Daily amount of food consumed per mouse at 2 and 6 months of age. (B) Quantification of blood cholesterol (Chol) and triglyceride (TG) levels at 2 and 6 months of age. Results are average ± SEM (n≥5), *p < 0.05. (DOCX) [file pone.0255687.s001.docx]

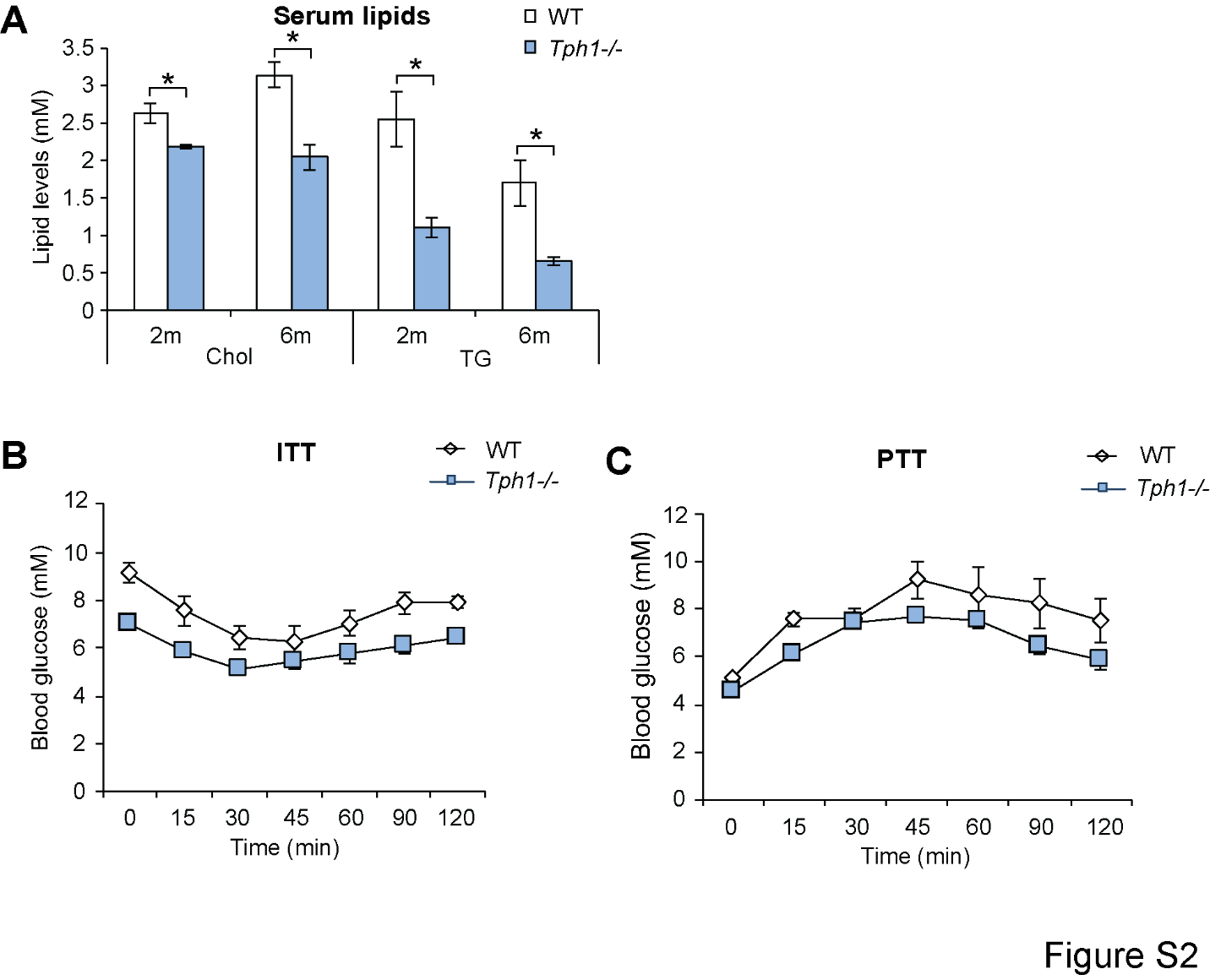

Supplement: S2 Fig — (A) Quantification of blood cholesterol (Chol) and triglyceride (TG) levels in wild type (WT) and Tph1 deficient (Tph1-/-) mice at 2 and 6 months of age. (B) Insulin tolerance test (ITT) in WT and Tph1-/- mice at 6 months of age. (C) Pyruvate tolerance test (PTT) in WT and Tph1-/- mice at 6 months of age. Results are average ± SEM (n≥5), *p < 0.05. (DOCX) [file pone.0255687.s002.docx]

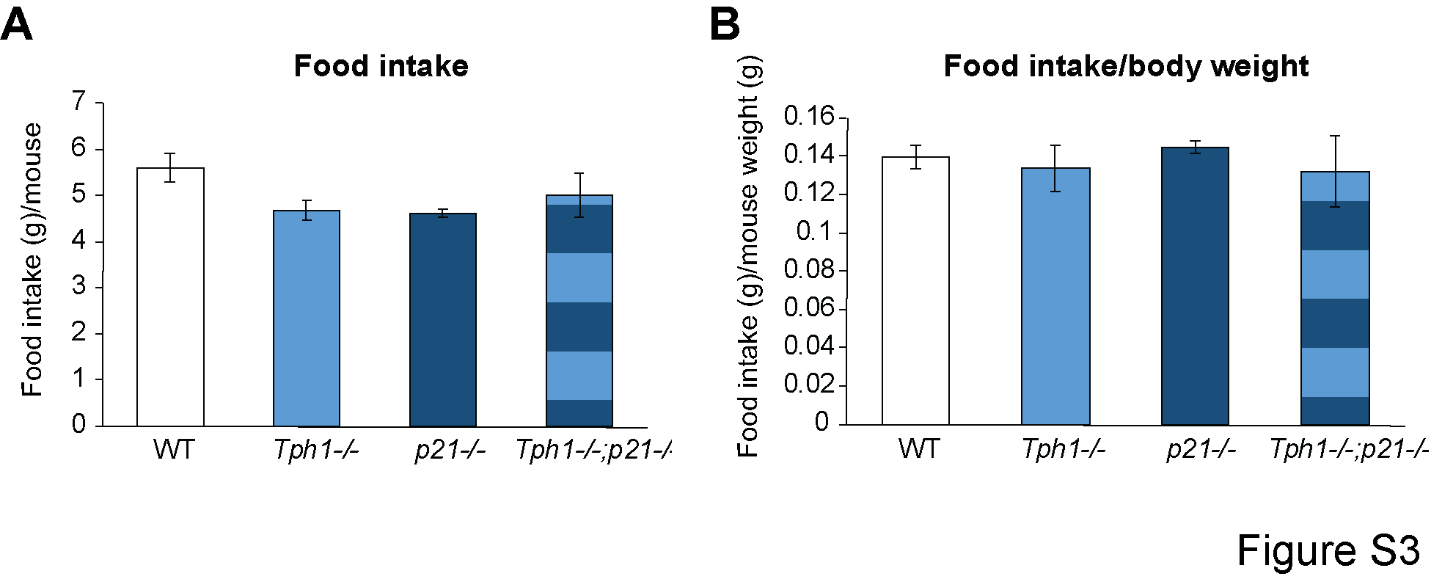

Supplement: S3 Fig — (A) Daily amount of food consumed per mouse in wild type (WT), Tph1 deficient (Tph1-/-), p21 deficient (p21-/-), and combined Tph1 and p21 deficient (Tph1-/-;p21-/-) mice at 6 months of age. (B) Daily amount of food consumed per mouse normalised on body weight in WT, Tph1-/-, p21-/-, and Tph1-/-;p21-/-) mice at 6 months of age. Results are average ± SEM (n≥5). (DOCX) [file pone.0255687.s003.docx]
